# Mapping of oxytocin‐ and arginine vasopressin‐expressing neurons with calbindin 1 or reelin in the male mouse brain

**DOI:** 10.1111/jne.70228

**Published:** 2026-07-06

**Authors:** Naranbat Nasanbuyan, Masahide Yoshida, Yuki Takayanagi, Keiichi Itoi, Tatsushi Onaka

**Affiliations:** ^1^ Division of Brain and Neurophysiology, Department of Physiology Jichi Medical University Shimotsuke Japan; ^2^ Graduate School of Medicine Tohoku University Sendai Japan

**Keywords:** arginine vasopressin, Calbindin 1, Corticotropin‐releasing hormone, oxytocin, Reelin

## Abstract

Oxytocin (OXT)‐ and arginine vasopressin (AVP)‐synthesizing neurons are traditionally classified into magnocellular and parvocellular types based on their projection targets. However, molecular markers that reliably distinguish these neuronal subtypes across hypothalamic regions remain undefined. Recent single‐cell transcriptomic studies suggest that *Calb1* and *Reln* are differentially expressed in magnocellular and parvocellular OXT‐synthesizing neurons in the hypothalamic paraventricular nucleus (PVN). Whether this molecular distinction extends to OXT neurons beyond the PVN or to AVP neurons has remained unclear. Fluorogold (FG) was administered to adult male C57BL/6J mice via tail vein injection. Calbindin 1 and reelin expression in OXT‐ and AVP‐synthesizing neurons in the bed nucleus of the stria terminalis (BNST), PVN, supraoptic nucleus (SON), and retrochiasmatic supraoptic nucleus (SOR) was examined by immunocytochemistry. FG‐labeled neurons were distributed in the PVN, SON, and SOR. Most OXT‐immunoreactive (ir) neurons in the anterior PVN, SON, and SOR were FG‐positive, whereas many OXT‐ir neurons in the posterior PVN were FG‐negative. Most FG‐positive OXT‐ir neurons showed calbindin 1 immunoreactivity. Two‐thirds of FG‐negative OXT‐ir neurons in the posterior PVN expressed reelin. Almost all Reln‐positive OXT‐ir neurons were FG‐negative. Nearly all AVP‐ir neurons were FG‐positive, and more than half expressed calbindin 1, whereas reelin expression was not detected. In corticotropin‐releasing hormone (CRH)‐Venus knock‐in mice, AVP‐ir neurons and CRH‐positive neurons were not colocalized within the PVN, and AVP‐ir and CRH‐positive fibers were localized to distinct layers of the median eminence. These findings indicate that AVP expression in parvocellular CRH neurons was below the detection threshold of the methods used in this study. Calbindin 1 serves as a marker for magnocellular OXT neurons, whereas reelin identifies a subset of parvocellular OXT neurons. Given that the detected AVP‐ir neurons were predominantly magnocellular, calbindin 1 labels a subset of this magnocellular AVP population. This molecular distinction provides a framework for distinguishing OXT‐ and AVP‐expressing neuronal populations and for examining their respective roles in endocrine and behavioral regulation.

## Graphical abstract

Calbindin 1 marks fluorogold‐positive magnocellular oxytocin neurons and a subset of magnocellular vasopressin neurons, whereas reelin marks a subset of fluorogold‐negative parvocellular oxytocin neurons.

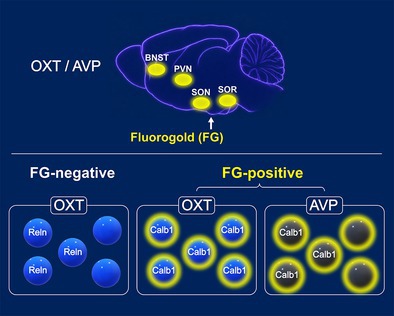

## 1 INTRODUCTION

Oxytocin (OXT) and arginine vasopressin (AVP) play critical roles in reproduction, social behavior, stress regulation, energy metabolism, and osmoregulation.^1^, ^2^, ^3^ OXT‐ and AVP‐synthesizing neurons have been classified into two subtypes, magnocellular and parvocellular, according to their projection targets. OXT‐ and AVP‐synthesizing neurons are located mainly in the hypothalamic paraventricular nucleus (PVN), supraoptic nucleus (SON), and retrochiasmatic supraoptic nucleus (SOR), and to a lesser extent in the bed nucleus of the stria terminalis (BNST).^4^, ^5^ Magnocellular OXT neurons project to the posterior pituitary to release OXT into the circulation and send collateral projections to the forebrain,^6^ whereas parvocellular OXT neurons innervate the forebrain, brainstem, and spinal cord.^7^, ^8^ In the AVP system, magnocellular AVP neurons are located mainly in the PVN and SON and project through the internal layer of the median eminence (ME) to the posterior pituitary, where they release AVP into the systemic circulation. By contrast, parvocellular AVP neurons coexpress corticotropin‐releasing hormone (CRH) in the PVN and project to the external layer of the ME, where they release AVP and CRH into the pituitary portal vessels to stimulate adrenocorticotropic hormone (ACTH) secretion from the anterior pituitary.^9^, ^10^, ^11^

Although single‐cell transcriptomic studies have revealed multiple genetically distinct OXT and AVP neuronal subtypes within the hypothalamus,^12^, ^13^ molecular criteria that reliably distinguish magnocellular and parvocellular OXT and AVP neurons across hypothalamic regions remain incompletely defined. Peripheral administration of Fluorogold (FG) has long served as a retrograde tracer to differentiate magnocellular from parvocellular OXT neurons, with FG‐positive cells classified as magnocellular and FG‐negative cells as parvocellular.^14^ By contrast, peripherally administered FG can label both magnocellular AVP neurons and parvocellular neuroendocrine AVP neurons via uptake at terminals in the posterior pituitary and ME, which are located outside the blood–brain barrier,^15^, ^16^ thereby limiting its ability to distinguish these AVP neuronal populations. Furthermore, intravenous FG injections are technically demanding and have been associated with neurotoxicity due to prolonged intracellular accumulation, reduction of c‐Fos expression, and transient alterations in axonal structure and nerve conduction.^17^, ^18^, ^19^ Therefore, identifying molecular markers that reliably distinguish magnocellular and parvocellular OXT and AVP neurons represents a valuable alternative to conventional tracing techniques.

Recent single‐cell RNA sequencing (scRNA‐seq) studies have identified several candidate molecules in the PVN, among which *Calb1* and *Reln* exhibit particularly distinct expression patterns. *Calb1* expression is enriched in magnocellular OXT neurons in the PVN, whereas *Reln* is expressed predominantly in parvocellular OXT‐synthesizing neurons.^20^, ^21^, ^22^ However, it remains unclear whether similar molecular distinctions apply to OXT neurons in other brain regions, such as the BNST, SON, and SOR. In addition, whether comparable molecular markers distinguish magnocellular and parvocellular AVP neurons has remained unresolved.

Here, we investigated whether calbindin 1 and reelin can serve as molecular markers to distinguish magnocellular and parvocellular OXT‐ and AVP‐synthesizing neurons in the BNST, PVN, SON, and SOR. We combined immunocytochemistry with FG retrograde tracing. Parvocellular AVP neurons in the PVN are known to coexpress CRH^23^, ^24^, ^25^ and expression of both AVP and CRH in these neurons is low and difficult to detect in normal animals using conventional immunocytochemical methods with commonly available antibodies.^26^, ^27^ We therefore used CRH‐Venus mice to identify CRH‐synthesizing neurons and to determine whether AVP‐ir neurons correspond to magnocellular or parvocellular populations based on their association with CRH expression and projections to distinct layers of the ME.

## 2 MATERIALS AND METHODS

### 2.1 Animals

Adult male C57BL/6J mice (The Jackson Laboratory Japan, Kanagawa, Japan) and CRH‐Venus knock‐in heterozygous mice^28^ were used. Mice were housed under a 12:12 h light: dark cycle (lights on 7:30 a.m. to 7:30 p.m.) at 22 ± 2°C and 40%–70% relative humidity. All mice were allowed a standard laboratory rodent diet and water ad libitum. All animal procedures were approved by the Institutional Animal Experiment Committee of Jichi Medical University and were conducted in accordance with the Institutional Regulations for Animal Experiments and Fundamental Guidelines for Proper Conduct of Animal Experiments and Related Activities in Academic Research Institutions under the authority of the Ministry of Education, Culture, Sports, Science and Technology of Japan.

### 2.2 Intravenous injection of Fluorogold

Fluorogold (Santa Cruz Biotechnology, catalog No. sc‐358883) was dissolved in sterile 0.9% saline to prepare a 4% solution. To determine a protocol for FG administration, three routes were tested: intraperitoneal, retro‐orbital, and tail vein injections at a dose of 20 mg/kg with an injection volume of 5 mL/kg. We used 15 male C57BL/6J mice (*n* = 5 per group) to test the three routes. For intraperitoneal injections, FG was administered while the mice were conscious. For retro‐orbital and tail vein injections, mice were anesthetized with isoflurane (4% in air for induction, 2% for maintenance; airflow rate, 1 L/min) and maintained under anesthesia through a nose nozzle while positioned on a heating pad. For retro‐orbital vein injections, FG was injected using a 32‐G needle. For tail vein injection, anesthetized mice were placed on a heating pad for 2–3 min to facilitate venous access, and the tail was cleaned with ethanol before FG was injected into the lateral tail vein using a 32 G needle. After injection, mice were returned to their home cages, and brains were collected 1 week later. Retro‐orbital and tail vein injections produced consistent FG labeling within the hypothalamic area, whereas intraperitoneal injection did not. Therefore, tail vein injection was used for the subsequent experiments.

A total of 4 male C57BL/6J mice received a tail vein injection of 4% FG for further analyses of calbindin 1 and reelin expression in OXT‐ and AVP‐synthesizing neurons. The validity of the retrograde labeling approach was confirmed by observing dense FG signals in circumventricular organs, which lack a blood–brain barrier, such as the subfornical organ and the vascular organ of the lamina terminalis.^29^, ^30^

### 2.3 Tissue preparation and immunocytochemistry

Mice were deeply anesthetized with an intraperitoneal injection of tribromoethanol (Fujifilm Wako Pure Chemical Corporation, Osaka, Japan) at a dose of 200 mg/kg and perfused transcardially with 0.9% sodium chloride containing heparin (20 U/L), followed by 4% paraformaldehyde (PFA) in 0.1 M phosphate buffer (PB; pH 7.4). Brains were removed and postfixed overnight in 4% PFA in 0.1 M PB, then cryoprotected by immersion in 0.1 M PB containing 30% sucrose at 4°C until they sank. The brains were subsequently frozen on dry ice and stored at −80°C until use. Sections were cut in the coronal plane at a thickness of 30 μm using a freezing sliding microtome. All brain sections were collected in 0.1 M PB, and every third section was further processed for immunocytochemical staining.

To examine the colocalization of OXT‐ and AVP‐synthesizing neurons with calbindin 1 or reelin, brain sections from FG‐injected male C57BL/6J mice were processed for immunocytochemistry. Nonspecific binding sites in the sections were first blocked with 0.1 M PB with 0.3% Triton X‐100 containing 10% normal donkey serum at room temperature for 60 min and then incubated at 4°C for 2 days with primary antibodies, guinea pig polyclonal anti‐oxytocin (Peninsula Laboratories, San Carlos, CA, USA; catalog No. T‐5021.0050; RRID: AB_518526) diluted 1:200,000 or guinea pig anti‐vasopressin (Peninsula Laboratories, catalog No. T‐5048.0050, RRID: AB_518680) diluted 1:50,000, together with either rabbit anti‐calbindin 1 (Cell Signaling Technology, Danvers, MA, USA; catalog No. 13176, RRID: AB_2687400) diluted 1:1000 or goat anti‐reelin (R&D Systems, Minneapolis, MN, USA; catalog No. AF3820, RRID: AB_2253745) diluted 1:1000 in 0.1 M PB containing 5% normal donkey serum and 0.3% Triton X‐100. After extensive washes, the sections were incubated at 4°C overnight with secondary antibodies, donkey anti‐guinea pig IgG Alexa Fluor 647 (Jackson ImmunoResearch, West Grove, PA, USA; catalog No. 706‐605‐148, RRID: AB_2340476) and either donkey anti‐rabbit IgG Alexa Fluor Plus 488 (Thermo Fisher Scientific; catalog No. A32790, RRID: AB_2762833) or donkey anti‐goat IgG Alexa Fluor Plus 488 (Thermo Fisher Scientific; catalog No. A32814, RRID: AB_2762838) in 0.1 M PB containing 5% normal donkey serum and 0.3% Triton X‐100. After extensive washes, the sections were mounted onto glass slides.

To examine the colocalization of AVP‐synthesizing neurons with calbindin 1 and CRH, brain sections from male CRH‐Venus knock‐in mice were processed for immunocytochemistry. The sections were first blocked with 0.1 M PB with 0.3% Triton X‐100 containing 10% normal donkey serum at room temperature for 60 min and then incubated at 4°C for 2 days with primary antibodies, guinea pig anti‐vasopressin diluted 1:50,000, rabbit anti‐calbindin 1 diluted 1:1000, and rat anti‐GFP antibody (Nacalai Tesque, Kyoto, Japan; catalog No. GF090R; RRID: AB_2314545) diluted 1:1000 in 0.1 M PB containing 0.3% Triton X‐100 and 5% normal donkey serum. After extensive washes, the sections were incubated at 4°C overnight with secondary antibodies, donkey anti‐guinea pig IgG Alexa Fluor 647, donkey anti‐rabbit IgG Alexa Fluor 546 (Thermo Fisher Scientific; catalog No. A10040, RRID: AB_2534016), and donkey anti‐rat IgG Alexa Fluor 488 (Thermo Fisher Scientific; catalog No. 21208, RRID: AB_2535794) in 0.1 M PB containing 5% normal donkey serum and 0.3% Triton X‐100. After extensive washes, the sections were mounted onto glass slides and counterstained with NucBlue (Thermo Fisher Scientific; catalog No. P36981).

The specificity of antibodies against OXT, AVP, and GFP has been confirmed in previous reports. The OXT antiserum (T‐5021) has minimal cross‐reactivity with (Arg^8^)‐vasopressin (manufacturer's data sheets). No immunostaining was observed in control sections in which the OXT antiserum was omitted or preadsorbed with 100 μM oxytocin at 4°C for 24 h before tissue incubation. Preadsorption with 100 μM (Arg^8^)‐vasopressin did not impair immunostaining.^31^ The AVP antiserum (T‐5048) has minimal cross‐reactivity with (Lys^8^)‐vasopressin or oxytocin, as determined by radioimmunoassay (manufacturer's data sheet). No immunostaining was observed in control sections in which the AVP antiserum was omitted, preadsorbed with 50 μM (Arg^8^)‐vasopressin at room temperature for 1 h before tissue incubation overnight, or in brain sections from vasopressin‐diphtheria toxin receptor‐expressing transgenic rats in which AVP‐expressing cells had been ablated by diphtheria toxin administration.^32^, ^33^ Preadsorption with 50 μM oxytocin did not affect immunostaining.^33^ The specificity of the GFP (GF090R) antibody was verified by the absence of immunoreactive signals in wild‐type mice.^34^ The specificity of the reelin antibody (AF3820) was verified by the absence of immunoblot signals in reelin‐deficient mice.^35^ The calbindin antibody (13176) has been well characterized by the manufacturer (according to the manufacturer's data sheets). For consistency with figure labeling, calbindin 1 and reelin are abbreviated as Calb1 and Reln, respectively, in figure panels and legends.

### 2.4 Image acquisition and analysis

#### 2.4.1 Quantification of FG signals

Images containing FG‐positive labeling were obtained at 40× magnification using a confocal microscope (Leica TCS SP5; Leica Microsystems, Wetzlar, Germany) equipped with Leica Application Suite X software. The BNST, PVN, SON, and SOR were identified using anatomical landmarks from the mouse brain atlas of Franklin and Paxinos.^36^ FG signals were quantified with ImageJ software (version 1.54k; National Institutes of Health, Bethesda, MD, USA) using the *Analyze Particles* function. All FG‐positive signals within a fixed area (390.8 μm × 390.8 μm) encompassing the nucleus of interest in each section were quantified, with signals smaller than 0.6 μm^2^ excluded from analysis. From brain sections containing the SON, the ventral meninges and associated vascular structures were removed manually during tissue processing to avoid inclusion of nonspecific FG signals. In the SOR, these regions were excluded from the analysis. The area of FG‐positive signals was measured separately in each hemisphere and summed to obtain bilateral values. The rostrocaudal range of analyzed sections was determined based on the distribution of FG labeling. For each animal, 4 BNST sections, 12 PVN sections, 10 SON sections, and 3 SOR sections were analyzed at 90 μm intervals. Sections were aligned across animals using corresponding anatomical landmarks to ensure comparison of equivalent rostrocaudal levels and consistent sampling of each nucleus.^36^ Values from corresponding sections were averaged across animals to obtain mean values for each section.

#### 2.4.2 Quantification of OXT‐ir, AVP‐ir, calbindin 1‐ir, reelin‐ir, and FG colocalization

For cellular colocalization analysis in the BNST, PVN, SON, and SOR, confocal images of cellular profiles labeled for OXT‐ir, AVP‐ir, calbindin 1‐ir, and reelin‐ir, together with FG‐positive labeling, were acquired using the same confocal microscope system. The BNST, PVN, SON, and SOR were identified using anatomical landmarks from the mouse brain atlas.^36^ All cellular profiles labeled for OXT‐ir or AVP‐ir within the image area encompassing each nucleus in both hemispheres were counted and then evaluated for colocalization with calbindin 1‐ir and FG or with reelin‐ir and FG. The rostrocaudal range of analyzed sections was determined based on the distribution of OXT‐ir or AVP‐ir cellular profile signals.

For analyses of OXT‐ir cellular profiles colocalized with calbindin 1‐ir and FG or with reelin‐ir and FG, 4 BNST sections, 10 PVN sections, 10 SON sections, and 3 SOR sections per animal were examined at 90 μm intervals.

For analyses of AVP‐ir cellular profiles colocalized with calbindin 1‐ir and FG or with reelin‐ir and FG, 6 PVN sections, 12 SON sections, and 3 SOR sections per animal were examined at 90 μm intervals.

For analyses of AVP‐ir cellular profiles colocalized with calbindin 1‐ir and CRH‐positive cells, 6 PVN sections per animal were examined at 90 μm intervals.

Sections were aligned across animals using corresponding anatomical landmarks, as described in the mouse brain atlas.^36^ Values from corresponding sections were averaged across animals to obtain mean values for each section. Data are presented as mean ± SEM. Data were visualized using GraphPad Prism (GraphPad Software, San Diego, CA, USA; version 10.3.1).

### 2.5 Declaration of generative AI and AI‐assisted technologies

AI tools were not used to generate data or interpret findings. In the writing process, the authors used ChatGPT, Copilot, and DeepL to refine the phrasing and ensure grammatical accuracy. The individual icons in the graphical abstract were partially generated using ChatGPT, and subsequently edited by the authors. All AI‐suggested revisions were critically evaluated and approved by the authors.

## 3 RESULTS

### 3.1 Distribution of Fluorogold‐labeled cells

**FIGURE 1:**
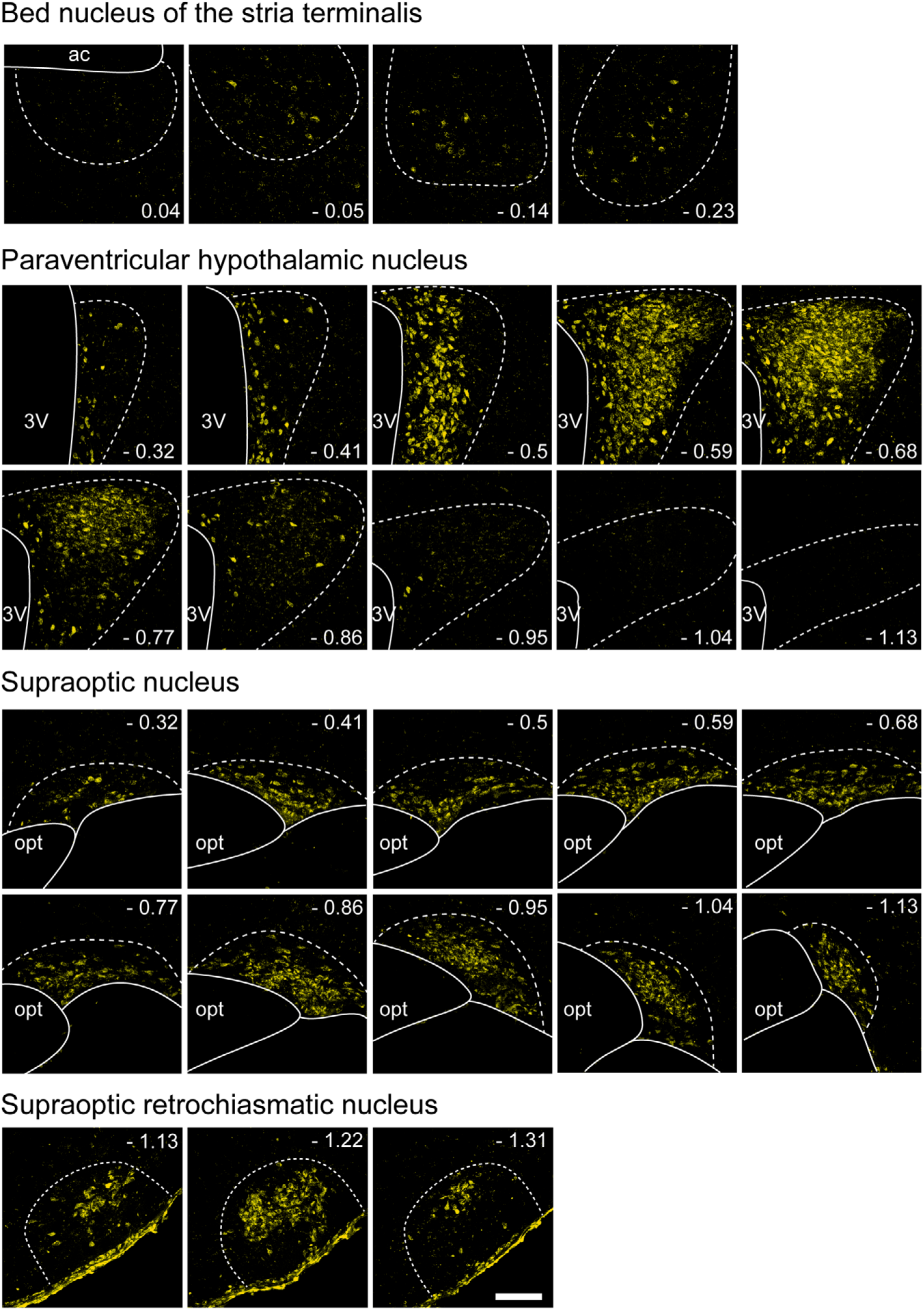
Distribution of tail vein‐injected Fluorogold in the brain of a male mouse. Confocal images showing retrogradely labeled Fluorogold‐positive cells. Fluorogold was injected intravenously via a lateral tail vein. Scale bar = 100 μm. Numbers in the corners indicate positions relative to bregma along the rostrocaudal axis. ac, anterior commissure; 3V, third ventricle; opt, optic tract.

**FIGURE 2:**
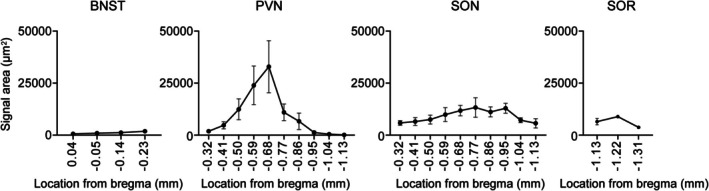
Quantification of tail vein‐injected Fluorogold distribution along the rostrocaudal axis. Data are presented as mean ± SEM (*n* = 4). For each animal, 4 sections of the BNST, 10 sections of the PVN, 10 sections of the SON, and 3 sections of the SOR were examined at 90 μm intervals. BNST, bed nucleus of the stria terminalis; PVN, paraventricular nucleus of the hypothalamus; SON, supraoptic nucleus; SOR, retrochiasmatic supraoptic nucleus.

We first examined the distribution of FG in brain sections obtained 1 week after injection, focusing on the BNST, PVN, SON, and SOR (Figures 1 and S1). Sparse FG labeling was observed in the BNST. In the PVN, FG labeling was dense in the anterior part and markedly reduced in the posterior part. FG‐positive cells were distributed throughout the SON and SOR along the rostrocaudal axis, with slightly higher abundance centrally (Figure 2).

### 3.2 Calbindin 1 and reelin expression in OXT‐ir neurons

**FIGURE 3:**
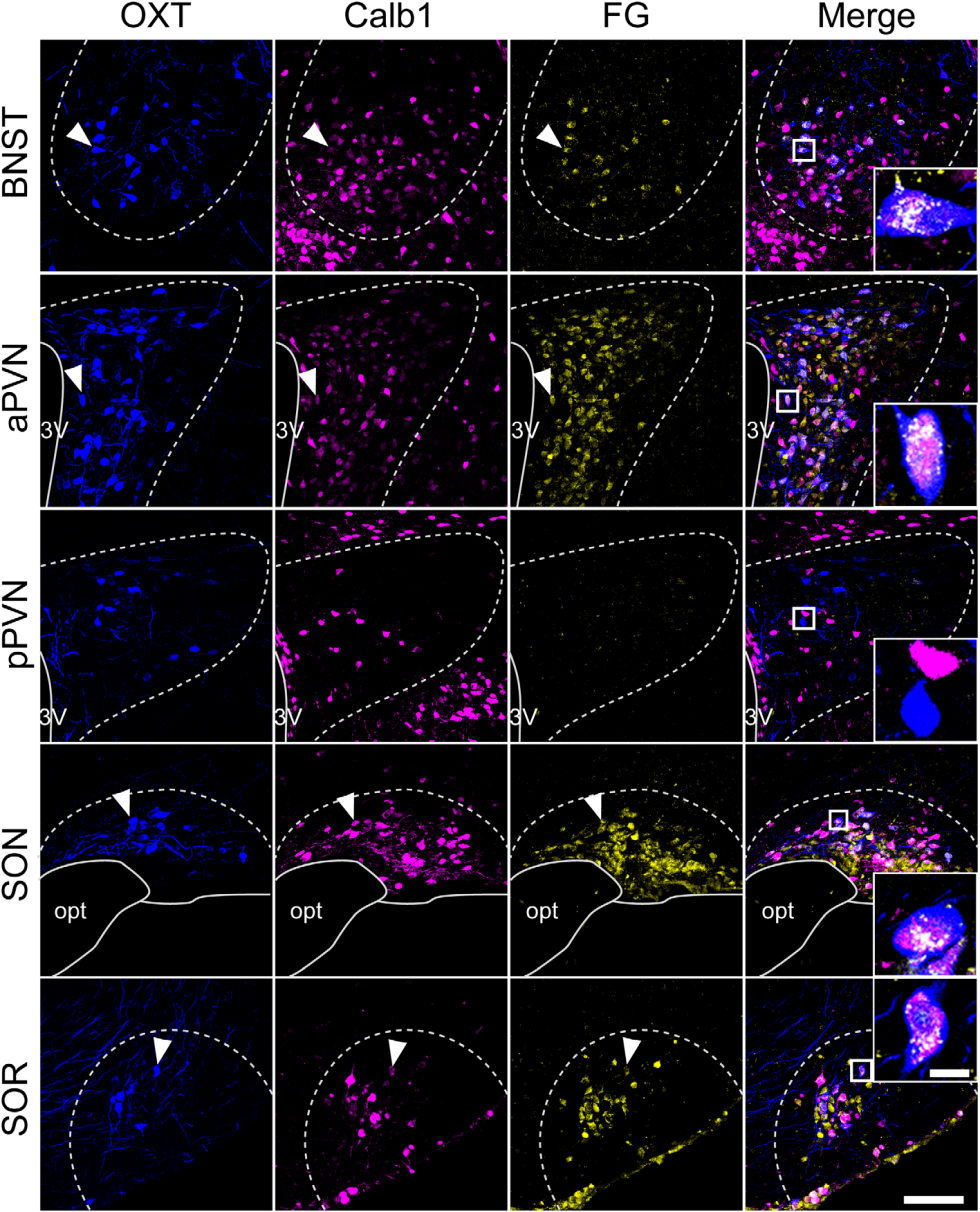
Distribution of oxytocin‐immunoreactive (OXT‐ir), calbindin 1‐immunoreactive (Calb1‐ir), and Fluorogold (FG)‐labeled neurons. Representative images of brain sections showing OXT‐ir, Calb1‐ir, and FG‐positive cells. Insets provide enlarged views of the regions outlined by squares in the low‐magnification images. Arrowheads and rectangles indicate cells shown at higher magnification in the insets. Scale bar = 100 μm. BNST, bed nucleus of the stria terminalis; PVN, paraventricular nucleus of the hypothalamus; aPVN, anterior PVN; pPVN, posterior PVN; SON, supraoptic nucleus; SOR, retrochiasmatic supraoptic nucleus; 3V, third ventricle; opt, optic tract.

**FIGURE 4:**
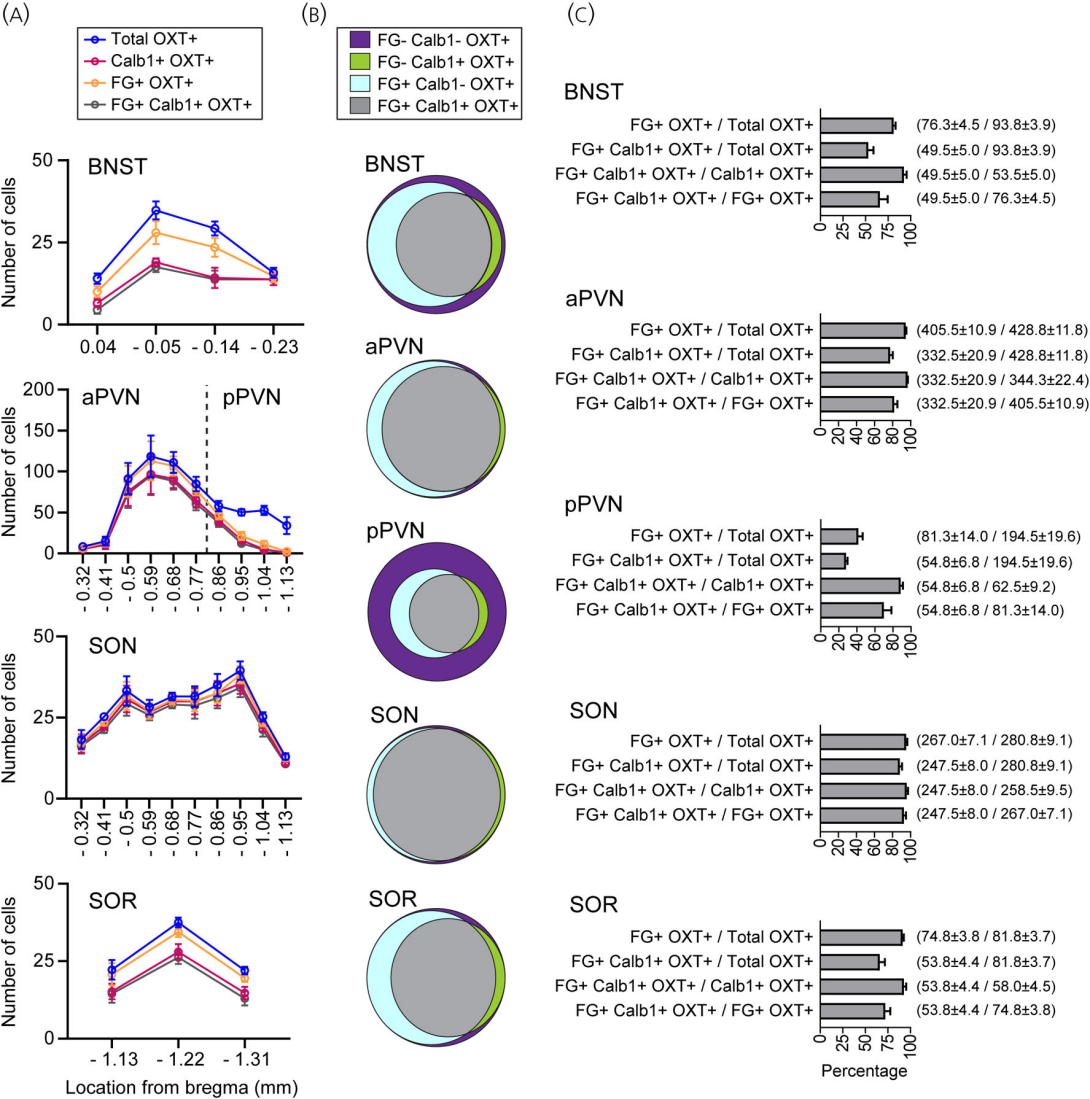
Quantification of oxytocin‐immunoreactive (OXT‐ir), calbindin 1‐immunoreactive (Calb1‐ir), and Fluorogold (FG)‐labeled neurons. (A) Distribution of total OXT‐ir cells, Calb1‐ir OXT‐ir cells, FG‐positive OXT‐ir cells, and FG‐positive Calb1‐ir OXT‐ir cells along the rostrocaudal axis. (B) Venn diagrams show the colocalization of Calb1‐ir and FG‐positive cells within the OXT‐ir cell population in each region. (C) Percentages of FG‐positive OXT‐ir cells relative to total OXT‐ir cells, FG‐positive Calb1‐ir OXT‐ir cells relative to total OXT‐ir cells, FG‐positive Calb1‐ir OXT‐ir cells relative to Calb1‐ir OXT‐ir cells, and FG‐positive Calb1‐ir OXT‐ir cells relative to FG‐positive OXT‐ir cells across the examined regions. Data are presented as mean ± SEM (*n* = 4). BNST, bed nucleus of the stria terminalis; PVN, paraventricular nucleus of the hypothalamus; aPVN, anterior PVN; pPVN, posterior PVN; SON, supraoptic nucleus; SOR, retrochiasmatic supraoptic nucleus.

The colocalization of FG and calbindin 1‐ir in OXT‐ir neurons was examined across the BNST, PVN, SON, and SOR (Figure 3). Over 80% of OXT‐ir neurons in the BNST (81.2 ± 3.0%), anterior PVN (94.6 ± 1.0%), SON (95.2 ± 1.7%), and SOR (91.4 ± 1.8%) were FG‐positive, suggesting that most OXT‐ir neurons in these areas are magnocellular (Figure 4). By contrast, only 41.6 ± 6.9% of OXT‐ir neurons in the posterior PVN were FG‐positive. The percentage of calbindin 1‐expressing OXT‐ir neurons among total OXT‐ir neurons was high in the BNST (57%), anterior PVN (80%), SON (92%), and SOR (71%), but was markedly lower in the posterior PVN (32%) (Table 1). Among FG‐positive OXT‐ir neurons, calbindin 1 expression was observed in the BNST (66.0 ± 12.1%), anterior PVN (81.8 ± 5.0%), posterior PVN (69.9 ± 11.8%), SON (92.7 ± 3.1%), and SOR (72.1 ± 7.9%). Conversely, most calbindin 1‐ir OXT‐ir neurons were FG‐positive in the BNST (92.5 ± 4.4%), anterior PVN (96.6 ± 0.5%), posterior PVN (88.5 ± 3.6%), SON (95.8 ± 2.0%), and SOR (92.8 ± 3.7%) (Figure 4). These results suggest that calbindin 1 serves as an immunocytochemical marker for magnocellular OXT‐synthesizing neurons in the BNST, anterior PVN, SON, and SOR.

**TABLE 1:** Calbindin 1 expression in OXT‐ir neurons.

Region		Total OXT‐ir (Cell number)	Calbindin 1‐ir OXT‐ir (Cell number)	Calbindin 1‐ir OXT‐ir/total OXT‐ir (%)
BNST		93.8 ± 3.9	53.5 ± 5.0	57.5 ± 6.5
PVN	Anterior	428.8 ± 11.9	344.3 ± 22.4	80.1 ± 3.2
	Posterior	194.5 ± 19.6	62.5 ± 9.2	31.8 ± 2.0
	Total	623.3 ± 27.3	406.8 ± 28.6	65.1 ± 2.5
SON		280.8 ± 9.1	258.5 ± 9.5	92.1 ± 2.2
SOR		81.6 ± 3.7	58.0 ± 4.6	71.1 ± 5.3

*Note*: PVN total represents the sum of anterior and posterior PVN values. Data are presented as mean ± SEM (*n* = 4).

Abbreviations: BNST, bed nucleus of the stria terminalis; PVN, paraventricular nucleus of the hypothalamus; SON, supraoptic nucleus; SOR, retrochiasmatic supraoptic nucleus; OXT‐ir, oxytocin‐immunoreactive; OXT‐ir neurons include both FG‐positive and FG‐negative cells.

**FIGURE 5:**
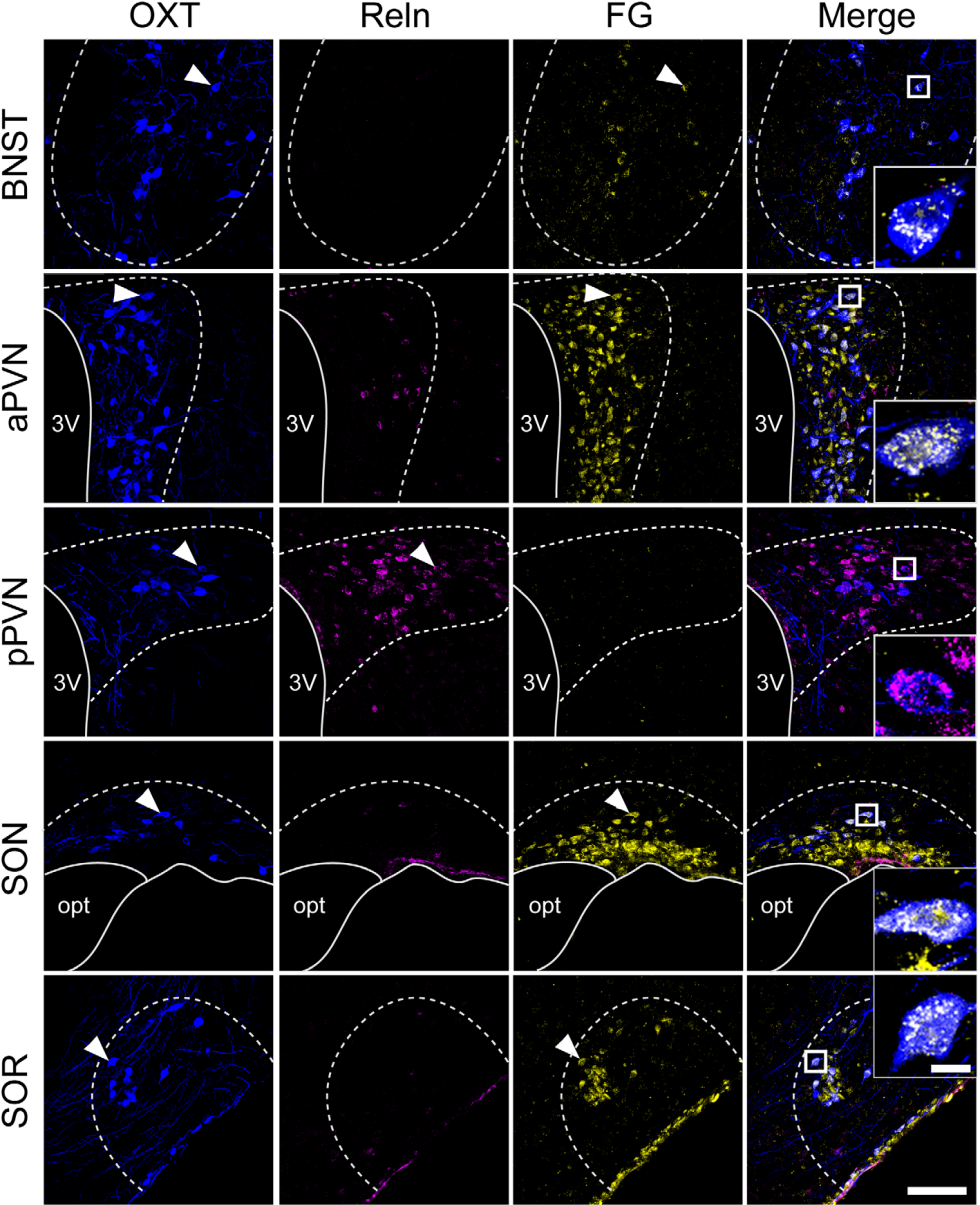
Distribution of oxytocin‐immunoreactive (OXT‐ir), reelin‐immunoreactive (Reln‐ir), and Fluorogold (FG)‐labeled neurons. Representative images of brain sections showing OXT‐ir, Reln‐ir, and FG‐positive cells. Insets provide enlarged views of the regions outlined by squares in the low‐magnification images. Arrowheads and rectangles indicate cells shown at higher magnification in the insets. Scale bar = 100 μm. BNST, bed nucleus of the stria terminalis; PVN, paraventricular nucleus of the hypothalamus; aPVN, anterior PVN; pPVN, posterior PVN; SON, supraoptic nucleus; SOR, retrochiasmatic supraoptic nucleus. 3V, third ventricle; opt, optic tract.

**FIGURE 6:**
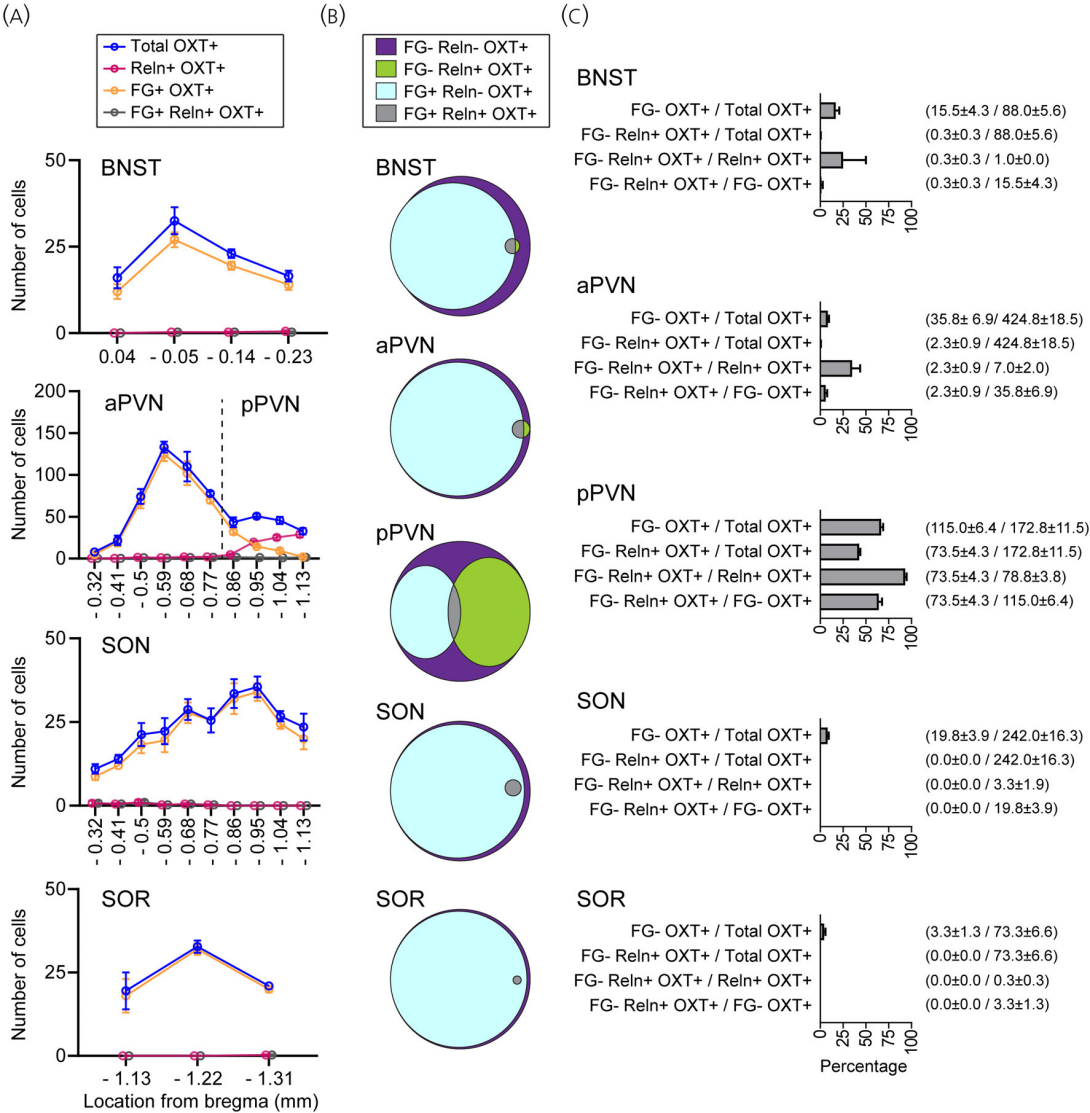
Quantification of oxytocin‐immunoreactive (OXT‐ir), reelin‐immunoreactive (Reln‐ir), and Fluorogold (FG)‐labeled neurons. (A) Distribution of total OXT‐ir cells, Reln‐ir OXT‐ir cells, FG‐positive OXT‐ir cells, and FG‐positive Reln‐ir OXT‐ir cells along the rostrocaudal axis. (B) Venn diagrams show the colocalization of Reln‐ir and FG‐positive cells within the OXT‐ir cell population in each region. (C) Percentages of FG‐negative OXT‐ir cells relative to total OXT‐ir cells, FG‐negative Reln‐ir OXT‐ir cells relative to total OXT‐ir cells, FG‐negative Reln‐ir OXT‐ir cells relative to Reln‐ir OXT‐ir cells, and FG‐negative Reln‐ir OXT‐ir cells relative to FG‐negative OXT‐ir cells across the examined regions. Data are presented as mean ± SEM (*n* = 4). BNST, bed nucleus of the stria terminalis; PVN, paraventricular nucleus of the hypothalamus; aPVN, anterior PVN; pPVN, posterior PVN; SON, supraoptic nucleus; SOR, retrochiasmatic supraoptic nucleus.

Reelin expression in OXT‐ir neurons was also examined (Figure 5). Reelin expression in all OXT‐ir neurons of the BNST, anterior PVN, SON, and SOR was minimal (<2%) but was distinctly higher in the posterior PVN (46%) (Table 2). Only a very small proportion of FG‐positive OXT‐ir neurons expressed reelin in the BNST (1.0 ± 0.4%), anterior PVN (1.2 ± 0.4%), posterior PVN (9.6 ± 2.1%), and SON (1.4 ± 0.8%), with only one cell detected in the SOR across 4 mice. FG‐negative OXT‐ir neurons expressing reelin were similarly rare in the BNST (1 cell across 4 mice) and anterior PVN (9 cells across 4 mice) and were absent in the SON and SOR. By contrast, the majority of FG‐negative OXT‐ir neurons in the posterior PVN expressed reelin (64.2 ± 4.9%), and most reelin‐ir OXT‐ir neurons lacked FG labeling (93.2 ± 2.0%) (Figure 6).

**TABLE 2:** Reelin expression in OXT‐ir neurons.

Region		Total OXT‐ir (Cell number)	Reelin‐ir OXT‐ir (Cell number)	Reelin‐ir OXT‐ir/Total OXT‐ir (%)
BNST		88.0 ± 5.6	1.0 ± 0.0	1.2 ± 0.1
PVN	Anterior	424.8 ± 18.5	7.0 ± 2.0	1.6 ± 0.4
	Posterior	172.8 ± 11.5	78.8 ± 3.8	45.8 ± 1.8
	Total	597.5 ± 26.5	85.8 ± 4.6	14.4 ± 0.5
SON		242.0 ± 16.3	3.3 ± 1.9	1.3 ± 0.7
SOR		73.3 ± 6.6	0.3 ± 0.3	0.3 ± 0.3

*Note*: OXT‐ir neurons include both FG‐positive and FG‐negative cells. PVN total represents the sum of anterior and posterior PVN values. Data are presented as mean ± SEM (*n* = 4).

Abbreviations: BNST, bed nucleus of the stria terminalis; PVN, paraventricular nucleus of the hypothalamus; SON, supraoptic nucleus; SOR, retrochiasmatic supraoptic nucleus; OXT‐ir, oxytocin‐immunoreactive.

Together, these findings indicate that FG‐positive, calbindin 1‐expressing magnocellular OXT neurons predominate in the BNST, anterior PVN, SON, and SOR, whereas FG‐negative, reelin‐expressing OXT neurons are localized mainly in the posterior PVN. These findings suggest that calbindin 1 can serve as a molecular marker for the magnocellular OXT population, whereas reelin labels a subset, rather than the entire population of parvocellular OXT neurons, indicating molecular heterogeneity within the parvocellular OXT population.

### 3.3 Calbindin 1 and reelin expression in AVP neurons

**FIGURE 7:**
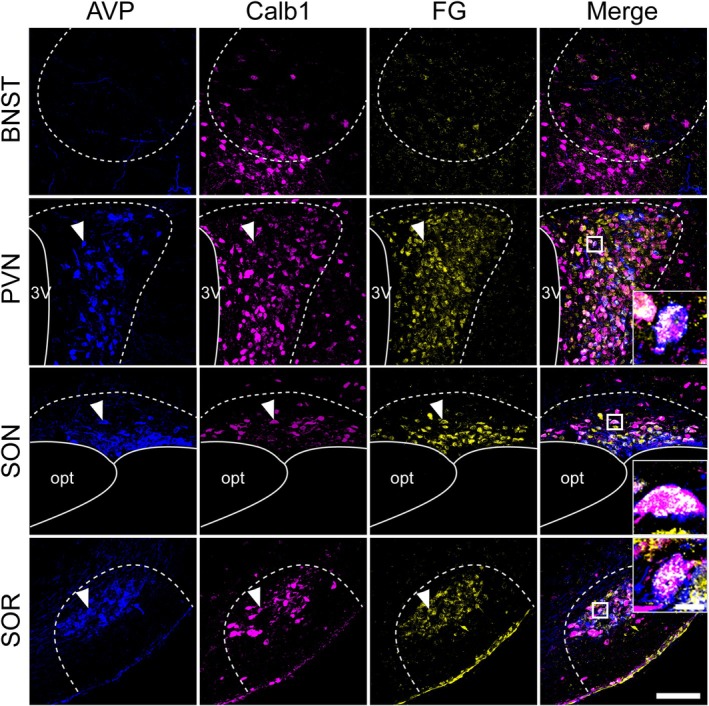
Distribution of arginine vasopressin‐immunoreactive (AVP‐ir), calbindin 1‐immunoreactive (Calb1‐ir), and Fluorogold (FG)‐labeled neurons. Representative images of brain sections showing AVP‐ir, Calb1‐ir, and FG‐positive cells. Insets provide enlarged views of the regions outlined by squares in the low‐magnification images. Arrowheads and rectangles indicate cells shown at higher magnification in the insets. Scale bar = 100 μm. BNST, bed nucleus of the stria terminalis; PVN, paraventricular nucleus of the hypothalamus; SON, supraoptic nucleus; SOR, retrochiasmatic supraoptic nucleus. 3V, third ventricle; opt, optic tract. No AVP‐ir cellular profiles were observed in the BNST.

**FIGURE 8:**
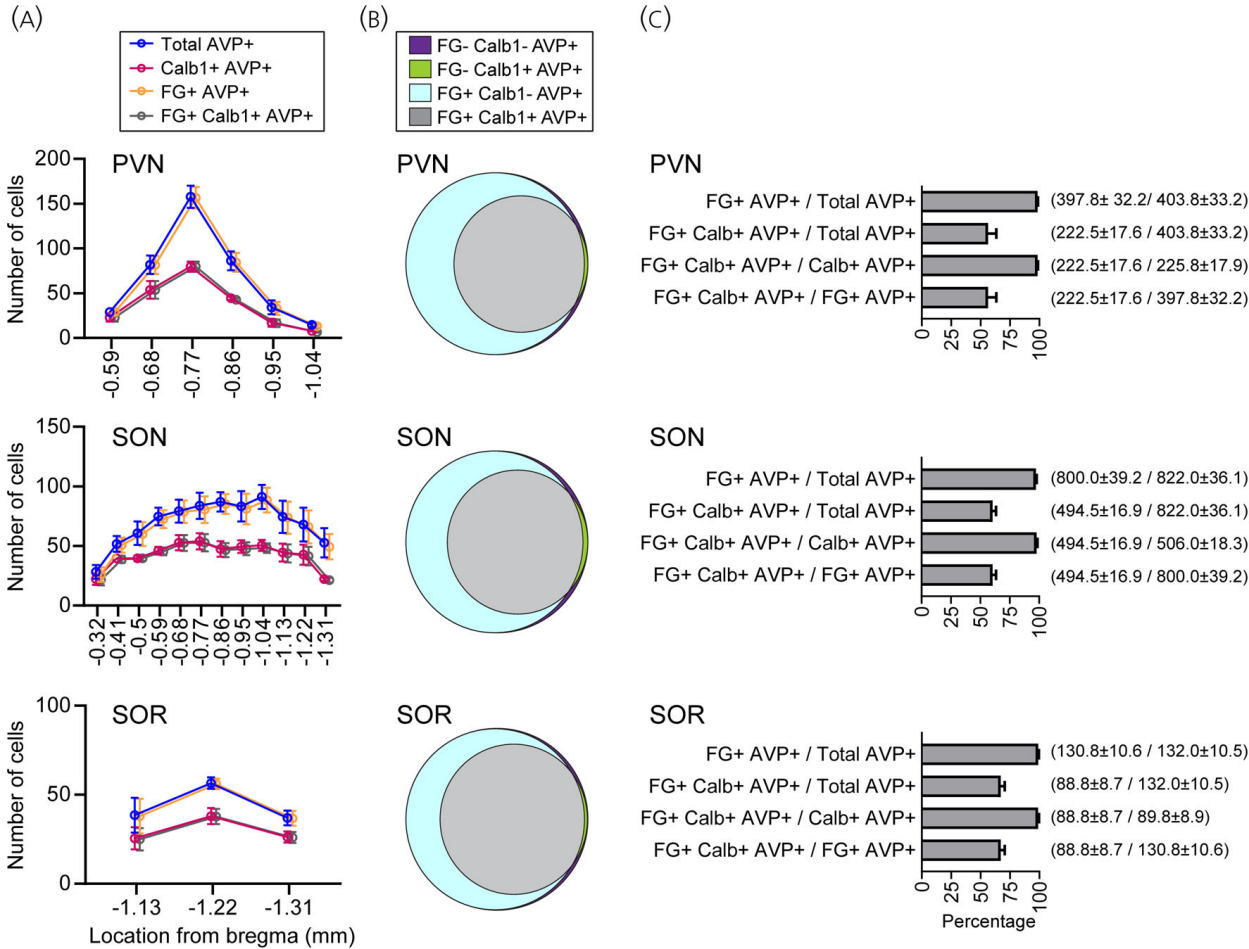
Quantification of arginine vasopressin‐immunoreactive (AVP‐ir), calbindin 1‐immunoreactive (Calb1‐ir), and Fluorogold (FG)‐labeled neurons. (A) Distribution of total AVP‐ir cells, Calb1‐ir AVP‐ir cells, FG‐positive AVP‐ir cells, and FG‐positive Calb1‐ir AVP‐ir cells along the rostrocaudal axis. (B) Venn diagrams show the colocalization of Calb1‐ir and FG‐positive cells within the AVP‐ir cell population in each region. (C) Percentages of FG‐positive AVP‐ir cells relative to total AVP‐ir cells, FG‐positive Calb1‐ir AVP‐ir cells relative to total AVP‐ir cells, FG‐positive Calb1‐ir AVP‐ir cells relative to Calb1‐ir AVP‐ir cells, and FG‐positive Calb1‐ir AVP‐ir cells relative to FG‐positive AVP‐ir cells across the examined regions. Data are presented as mean ± SEM (*n* = 4). BNST, bed nucleus of the stria terminalis; PVN, paraventricular nucleus of the hypothalamus; SON, supraoptic nucleus; SOR, retrochiasmatic supraoptic nucleus.

**FIGURE 9:**
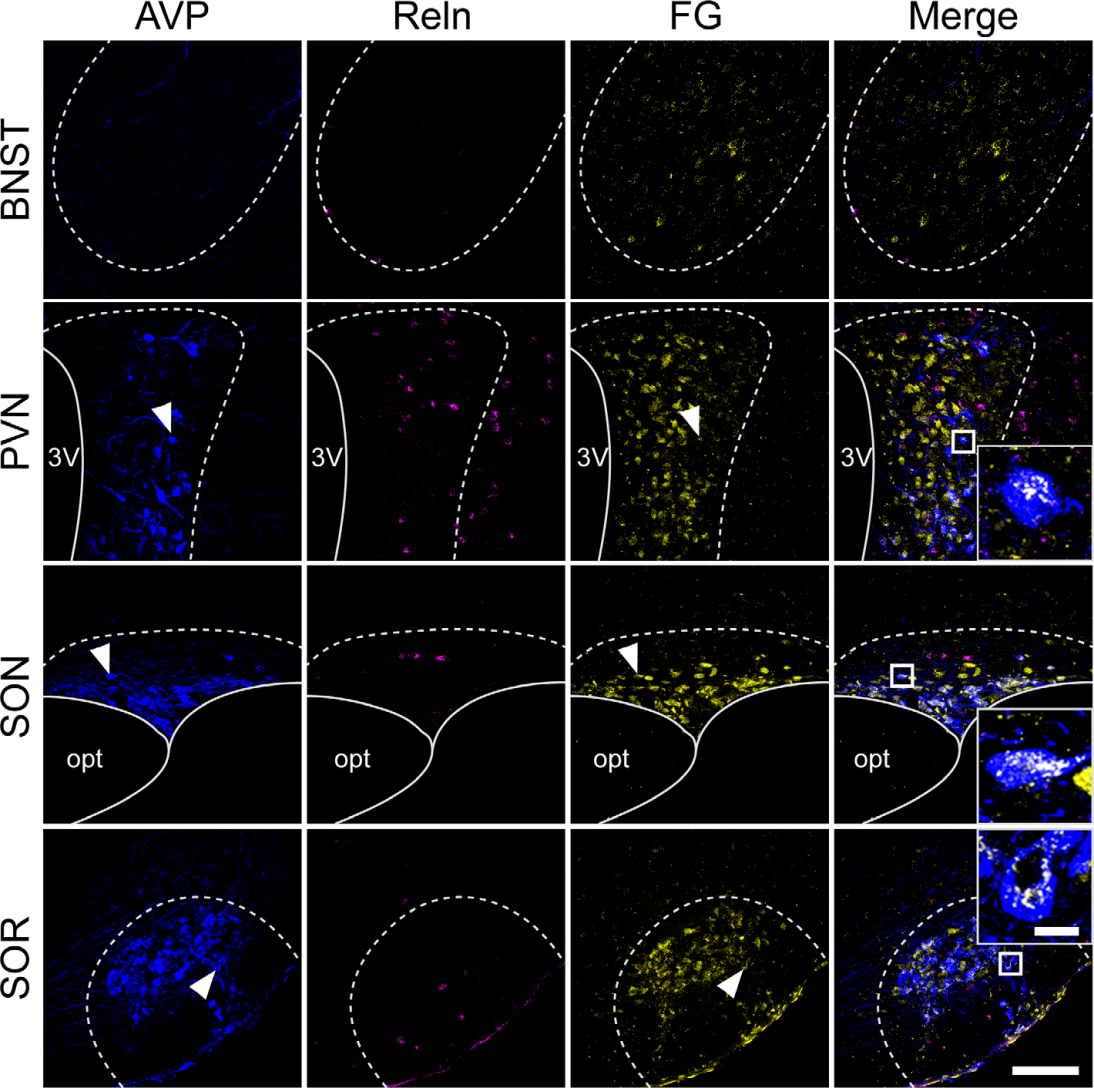
Distribution of arginine vasopressin‐immunoreactive (AVP‐ir), reelin‐immunoreactive (Reln‐ir), and Fluorogold (FG)‐labeled neurons. Representative images of brain sections showing AVP‐ir, Reln‐ir, and FG‐positive cells. Insets provide enlarged views of the regions outlined by squares in the low‐magnification images. Arrowheads and rectangles indicate cells shown at higher magnification in the insets. Scale bar = 100 μm. BNST, bed nucleus of the stria terminalis; PVN, paraventricular nucleus of the hypothalamus; SON, supraoptic nucleus; SOR, retrochiasmatic supraoptic nucleus. 3V, third ventricle; opt, optic tract. No AVP‐ir cellular profiles were observed in the BNST.

**FIGURE 10:**
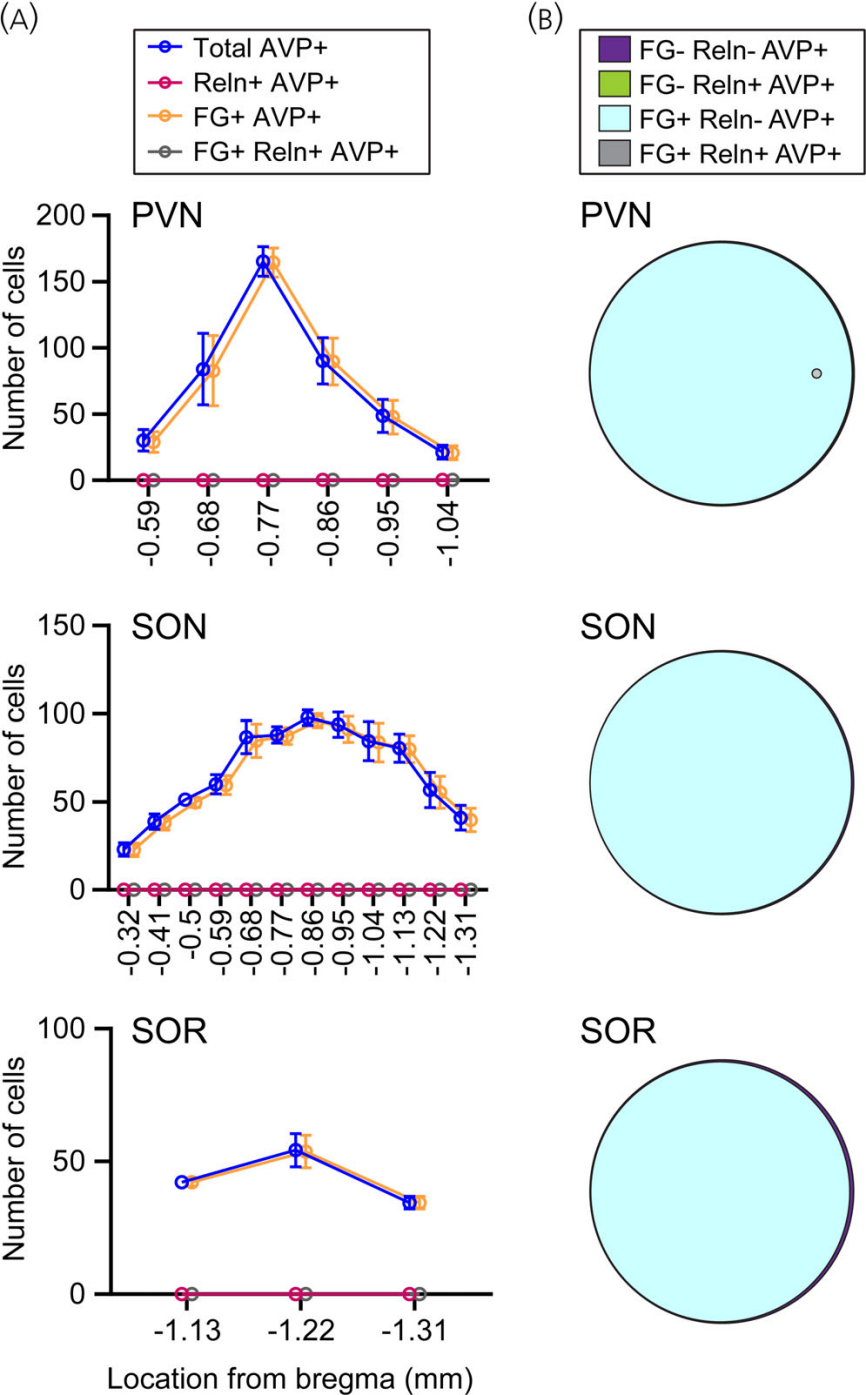
Quantification of arginine vasopressin‐immunoreactive (AVP‐ir), reelin‐immunoreactive (Reln‐ir), and Fluorogold (FG)‐labeled neurons. (A) Distribution of total AVP‐ir cells, Reln‐ir AVP‐ir cells, FG‐positive AVP‐ir cells, and FG‐positive Reln‐ir AVP‐ir cells along the rostrocaudal axis. (B) Venn diagrams show the colocalization of Reln‐ir and FG‐positive cells within the AVP‐ir cell population in each region. Reln‐ir AVP‐ir cells were rare across the examined brain regions. Data are presented as mean ± SEM (*n* = 4). BNST, bed nucleus of the stria terminalis; PVN, paraventricular nucleus of the hypothalamus; SON, supraoptic nucleus; SOR, retrochiasmatic supraoptic nucleus.

The colocalization of FG and calbindin 1‐ir in AVP‐ir neurons was examined across the BNST, PVN, SON, and SOR (Figure 7). No AVP‐ir cellular profiles were observed in the BNST. Across the PVN, SON, and SOR, 57%–68% of all AVP‐ir neurons coexpressed calbindin 1 (Table 3). Nearly all AVP‐ir cells were FG‐positive in the PVN (98.5 ± 0.4%), SON (97.2 ± 1.0%), and SOR (99.0 ± 0.8%). Among FG‐positive AVP‐ir neurons, more than half expressed calbindin 1 in the PVN (56.4 ± 9.9%), SON (60.4 ± 4.3%), and SOR (67.1 ± 4.9%). Conversely, nearly all calbindin 1‐ir AVP‐ir neurons were FG‐positive in the PVN (98.6 ± 0.3%), SON (97.8 ± 1.1%), and SOR (98.9 ± 1.0%) (Figure 8). Among all AVP‐ir neurons, reelin expression was detected in only two cells in the PVN and was absent in the SON and SOR across 4 mice (Table 4 and Figures 9 and 10).

**TABLE 3:** Calbindin 1 expression in AVP‐ir neurons.

Region	Total AVP‐ir (Cell number)	Calbindin 1‐ir AVP‐ir (Cell number)	Calbindin 1‐ir AVP‐ir/Total AVP‐ir (%)
PVN	403.8 ± 33.2	225.8 ± 17.9	57.2 ± 7.1
SON	822.0 ± 36.1	506.0 ± 18.4	61.9 ± 3.6
SOR	132.0 ± 10.5	89.8 ± 8.9	67.9 ± 3.9

*Note*: AVP‐ir neurons include both FG‐positive and FG‐negative cells. Data are presented as mean ± SEM (*n* = 4).

Abbreviations: PVN, paraventricular nucleus of the hypothalamus; SON, supraoptic nucleus; SOR, retrochiasmatic supraoptic nucleus; AVP‐ir, arginine vasopressin‐immunoreactive.

**TABLE 4:** Reelin expression in AVP‐ir neurons.

Region	Total AVP‐ir (Cell number)	Reelin‐ir AVP‐ir (Cell number)	Reelin‐ir AVP‐ir/Total AVP‐ir (%)
PVN	439.8 ± 29.2	0.5 ± 0.3	0.1 ± 0.1
SON	802.0 ± 39.7	0.0 ± 0.0	0.0 ± 0.0
SOR	131.0 ± 4.7	0.0 ± 0.0	0.0 ± 0.0

*Note*: AVP‐ir neurons include both FG‐positive and FG‐negative cells. Data are presented as mean ± SEM (*n* = 4).

Abbreviations: PVN, paraventricular nucleus of the hypothalamus; SON, supraoptic nucleus; SOR, retrochiasmatic supraoptic nucleus; AVP‐ir, arginine vasopressin‐immunoreactive.

**FIGURE 11:**
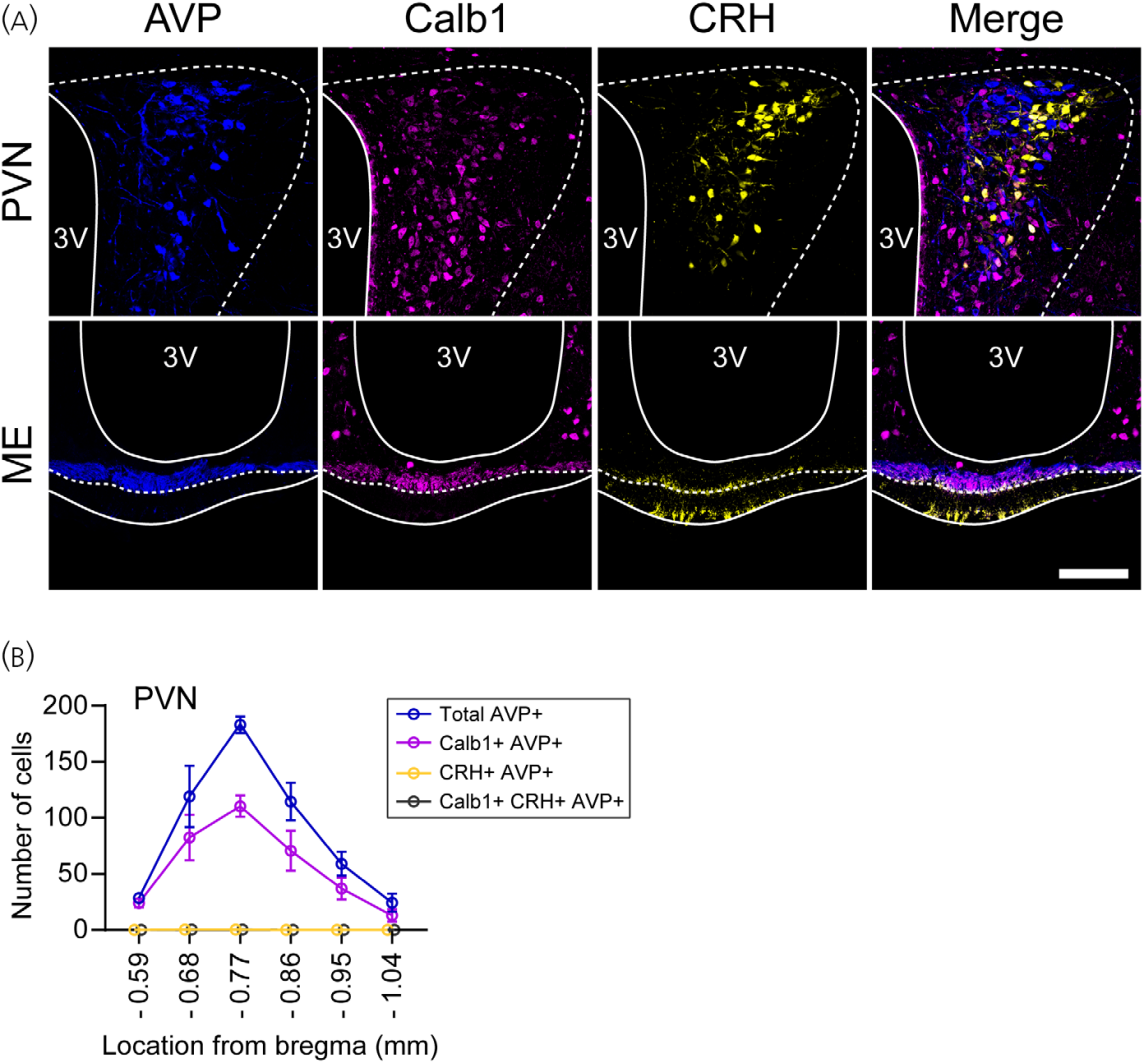
Distribution of arginine vasopressin‐immunoreactive (AVP‐ir), calbindin 1‐immunoreactive (Calb1‐ir), and corticotropin‐releasing hormone (CRH)‐positive cells in CRH‐Venus knock‐in mice. (A) Representative confocal images showing AVP‐ir, Calb1‐ir, and CRH‐positive cells in the PVN (top) and axonal fibers in the ME (bottom). The dotted line indicates the boundary between the inner and outer layers of the ME. Scale bar = 100 μm. (B) Distribution of total AVP‐ir cells, Calb1‐ir AVP‐ir cells, CRH‐positive AVP‐ir cells, and Calb1‐ir CRH‐positive AVP‐ir cells along the rostrocaudal axis of the PVN. Data are presented as mean ± SEM (*n* = 3). PVN, paraventricular nucleus of the hypothalamus; ME, median eminence; 3V, third ventricle.

Because parvocellular CRH‐synthesizing neurosecretory neurons are known to coexpress AVP,^23^, ^24^, ^25^ we assessed whether AVP expression in CRH neurons could be detected using the method employed in this study. In male CRH‐Venus knock‐in mice, CRH expression was not observed in the SON and SOR, whereas AVP‐ir neurons were detected in these regions. In the PVN, both AVP‐ir neurons and CRH‐expressing neurons were detected; however, no colocalization was observed (Figure 11A, top), indicating that the methods used here are not capable of detecting AVP in parvocellular neurosecretory neurons. In the ME, AVP‐ir fibers were localized to the internal layer, whereas CRH‐positive terminals were confined to the external layer (Figure 11A, bottom). Calbindin 1 was expressed in 63.6 ± 5.2% of all AVP‐ir neurons in the PVN, and colocalization between calbindin 1 and CRH was not observed (Figure 11B).

Moreover, the presence of calbindin 1‐ir in more than half of FG‐positive AVP‐ir neurons suggests that calbindin 1 labels a subset, rather than the entire population of magnocellular AVP neurons, indicating molecular heterogeneity within this population.

## 4 DISCUSSION

Here, we examined whether calbindin 1‐ir and reelin‐ir can distinguish between magnocellular and parvocellular OXT‐ and AVP‐synthesizing neurons. Most FG‐positive OXT‐ir neurons in the BNST, PVN, SON, and SOR express calbindin 1. By contrast, a subset of FG‐negative OXT‐ir neurons in the posterior PVN expresses reelin, indicating distinct molecular identities between magnocellular and parvocellular OXT neurons, consistent with previous reports.^20^, ^21^, ^22^ Nearly all AVP‐ir neurons detected in the PVN, SON, and SOR were FG‐positive, and more than half of these expressed calbindin 1, whereas reelin was not observed in AVP‐ir neurons. These findings suggest that calbindin 1 serves as a marker for magnocellular OXT neurons and that calbindin 1 and reelin can identify subsets of magnocellular AVP and parvocellular OXT neurons, respectively. However, neither molecule labels the entire population, and calbindin 1 identifies only a subset of magnocellular AVP neurons.

Previous immunocytochemical studies in rats showed predominant calbindin 1 detection in OXT neurons and its presence in a subset of AVP neurons in the SON.^37^, ^38^ In addition, reelin‐positive PVN cells colocalized mainly with OXT in rats, compared with AVP‐ and CRH‐positive neurons.^39^ Single‐cell RNA sequencing studies have further demonstrated differential expression of calbindin 1 and reelin in OXT neurons of the PVN in mice.^20^, ^21^, ^22^ Our findings extend this molecular distinction to the BNST, SON, and SOR. In all these nuclei, calbindin 1‐positive OXT‐ir neurons show strong overlap with FG labeling, whereas FG‐negative OXT‐ir neurons are scarce, suggesting that most OXT neurons in these nuclei are likely magnocellular, consistent with previous findings in rats.^6^ Previous studies in mice and rats have shown that OXT‐positive fibers are predominantly localized to the internal layer of the ME, through which magnocellular neurons project to the posterior pituitary,^4^, ^40^ whereas few fibers were present in the external layer of the ME. These observations support the interpretation that the FG‐positive OXT‐ir neurons observed in the present study project to the posterior pituitary. Our results show that calbindin 1‐positive OXT neurons colocalize with FG in the BNST, PVN, SON, and SOR, suggesting that these neurons constitute the magnocellular neurosecretory population of the hypothalamoneurohypophysial system. By contrast, reelin‐positive OXT‐ir neurons are located largely in the posterior PVN and were not labeled with FG, confirming that reelin is expressed in parvocellular OXT‐ir neurons in the posterior PVN, but not in the entire parvocellular OXT population.

Calbindin 1 expression was consistently high in FG‐positive magnocellular OXT‐ir neurons. By contrast, a minor proportion of FG‐positive OXT‐ir neurons were calbindin 1‐negative in the BNST, anterior PVN, posterior PVN, and SOR. Among FG‐positive AVP‐ir neurons, more than half were calbindin 1‐positive, whereas the remaining cells lacked calbindin 1 immunoreactivity. Consistently, recent single‐cell transcriptomic studies have identified multiple AVP neuronal subclusters in the PVN expressing *Tac1* and *Th*, which were suggested to be associated with magnocellular neurosecretory populations.^12^ Among these genes, *Tac1* may help further characterize FG‐positive, calbindin 1‐negative AVP neurons in future studies. Collectively, these findings suggest that magnocellular OXT and AVP neurons exhibit molecular heterogeneity in calbindin 1 expression. Calbindin 1 has been reported to regulate neuronal excitability by finely tuning intracellular Ca^2+^ buffering, preventing excessive Ca^2+^ accumulation during repetitive activity and thereby maintaining continuous firing dynamics.^40^, ^41^, ^42^ Electrophysiological studies have shown that magnocellular AVP neurons display phasic or continuous burst activity^43^, ^44^ and that calbindin 1 modulates the firing patterns.^45^ Whether firing patterns differ between calbindin 1‐positive and calbindin 1‐negative magnocellular neurons remains to be determined. By contrast, in the present study, the majority of the parvocellular FG‐negative OXT neurons within the posterior PVN expressed reelin. Parvocellular OXT neurons have been shown to express either glutamate or γ‐aminobutyric acid.^13^ Reelin functions as a synaptic modulator, enhancing *N*‐methyl‐d‐aspartate receptor signaling and promoting synaptic plasticity.^46^ Reelin expression in FG‐negative OXT neurons within the posterior PVN may contribute to the capacity of these parvocellular neurons to modulate central circuits governing behavior, energy metabolism, and autonomic regulation in response to internal or external environmental challenges.^47^, ^48^, ^49^ This molecular distinction provides a basis for understanding these different neuronal populations, comprising at least 4 types (FG‐positive neurons either expressing or lacking calbindin 1, and FG‐negative neurons either expressing or lacking reelin). Further research is necessary to clarify how these neuronal populations contribute to peripheral endocrine regulation and central functions.

In the present study, AVP immunoreactivity was not detected in the CRH‐expressing parvocellular neurons in the PVN. Single‐cell transcriptomic studies have identified a parvocellular cluster enriched for both AVP and CRH expression.^50^ The absence of AVP‐ir in CRH cells can be explained by the possibility that AVP levels are not sufficiently high to be detected in CRH‐expressing parvocellular neurons under the present experimental conditions. Therefore, it is likely that the AVP‐ir neurons identified here in the PVN represent predominantly magnocellular populations. Developmentally, parvocellular AVP and CRH neurons share common transcription factors, including Otp, Sim1, and Brn2, which direct neuroendocrine differentiation in the PVN.^51^ A single‐cell transcriptomic study showed that AVP‐ and CRH‐coexpressing cells are enriched in stress‐ and HPA‐axis‐related genes (*Crhr1, Nr3c1*) as well as genes related to neuropeptide processing and signaling (*Pcsk1n*, *Nnat*, and *Rgs2*), whereas *Calb1* is absent.^12^ These genes might serve as candidate markers for parvocellular AVP neurons, and their examination as such is warranted.

Here, AVP‐ir cellular profiles were not observed in the BNST. However, previous studies have demonstrated the presence of AVP‐expressing neurons in this region.^33^, ^52^, ^53^ AVP expression in the BNST is also likely to have been below the limit of detection of the immunohistochemical methods used in the present study.

Our findings provide molecular and anatomical evidence that calbindin 1 expression identifies the magnocellular OXT population and labels a subset of the magnocellular AVP population, whereas reelin expression is confined to a subset of the parvocellular OXT population. This molecular distinction provides a useful framework for characterizing distinct OXT and AVP neuronal populations, comprising at least 4 subtypes: FG‐positive neurons that express or lack calbindin 1, and FG‐negative neurons that express or lack reelin. This framework provides a basis for future studies investigating how distinct OXT and AVP populations differentially regulate endocrine and behavioral functions.

## AUTHOR CONTRIBUTIONS

**Keiichi Itoi:** Resources. **Masahide Yoshida:** Conceptualization; methodology; investigation; writing – review and editing; funding acquisition. **Naranbat Nasanbuyan:** Conceptualization; methodology; investigation; writing – original draft; writing – review and editing; funding acquisition. **Tatsushi Onaka:** Conceptualization; methodology; investigation; writing – original draft; writing – review and editing; funding acquisition. **Yuki Takayanagi:** Methodology; investigation; writing – review and editing; funding acquisition.

## CONFLICT OF INTEREST STATEMENT

The authors report no financial interests or potential conflicts of interest that could be perceived as prejudicing the impartiality of the research reported.

## Supporting information


**FIGURE S1.** Anatomical reference maps of coronal sections encompassing the BNST, PVN, SON, and SOR.
Schematic coronal drawings of the mouse brain indicate the anatomical locations of the BNST, PVN, SON, and SOR used for region identification and section alignment. Section levels correspond to the rostrocaudal coordinates shown in the upper left corner of each panel, based on the mouse brain atlas. Red rectangles indicate the fields of view used for imaging. Red dotted outlines delineate the nucleus of interest. BNST, bed nucleus of the stria terminalis; PVN, paraventricular nucleus of the hypothalamus; SON, supraoptic nucleus; SOR, retrochiasmatic supraoptic nucleus; ac, anterior commissure; 3V, third ventricle; opt, optic tract.

## Data Availability

The data that support the findings of this study are available from the corresponding author upon reasonable request.
