# Supplementary figures and images for: Mapping of oxytocin‐ and arginine vasopressin‐expressing neurons with calbindin 1 or reelin in the male mouse brain
Source: J Neuroendocrinol. 2026 Jul 6;38(7):e70228. doi: 10.1111/jne.70228 (PMC13337333; doi:10.1111/jne.70228)

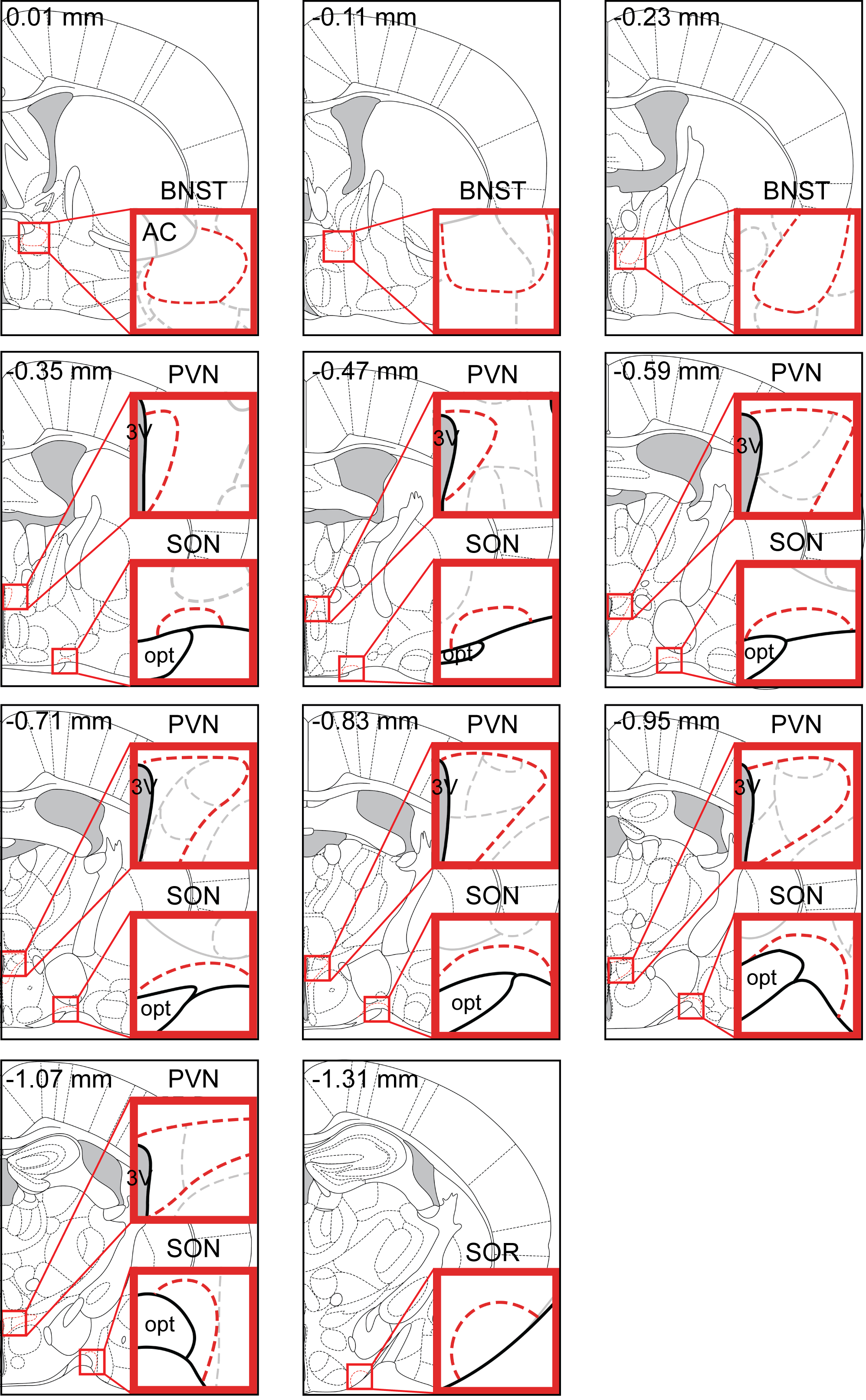

Supplement: Supplementary file 1 — FIGURE S1. Anatomical reference maps of coronal sections encompassing the BNST, PVN, SON, and SOR. Schematic coronal drawings of the mouse brain indicate the anatomical locations of the BNST, PVN, SON, and SOR used for region identification and section alignment. Section levels correspond to the rostrocaudal coordinates shown in the upper left corner of each panel, based on the mouse brain atlas. Red rectangles indicate the fields of view used for imaging. Red dotted outlines delineate the nucleus of interest. BNST, bed nucleus of the stria terminalis; PVN, paraventricular nucleus of the hypothalamus; SON, supraoptic nucleus; SOR, retrochiasmatic supraoptic nucleus; ac, anterior commissure; 3V, third ventricle; opt, optic tract. [file JNE-38-e70228-s001.tif]
